# Impact of Enhanced *Staphylococcus* DNA Extraction on Microbial Community Measures in Cystic Fibrosis Sputum

**DOI:** 10.1371/journal.pone.0033127

**Published:** 2012-03-08

**Authors:** Jiangchao Zhao, Lisa A. Carmody, Linda M. Kalikin, Jun Li, Joseph F. Petrosino, Patrick D. Schloss, Vincent B. Young, John J. LiPuma

**Affiliations:** 1 Departments of Pediatrics and Communicable Disease, University of Michigan, Ann Arbor, Michigan, United States of America; 2 Department of Human Genetics, University of Michigan, Ann Arbor, Michigan, United States of America; 3 Alkek Center for Metagenomics and Microbiome Research, Baylor College of Medicine, Houston, United States of America; 4 Department of Molecular Virology and Microbiology, Baylor College of Medicine, Houston, Texas, United States of America; 5 Department of Microbiology and Immunology, University of Michigan, Ann Arbor, Michigan, United States of America; 6 Internal Medicine/Infectious Diseases Division, University of Michigan, Ann Arbor, Michigan, United States of America; 7 Department of Epidemiology, University of Michigan, Ann Arbor, Michigan, United States of America; Louisiana State University and A & M College, United States of America

## Abstract

*Staphylococcus aureus* is a common constituent of the bacterial community inhabiting the airways of persons with cystic fibrosis (CF). Culture-independent studies have shown that this species is often present in relatively high abundance and would therefore be expected to exert a pronounced effect on measures of CF airway bacterial community structure. We investigated the impact of DNA extraction method on pyrosequencing-based measures of *Staphylococcus* abundance and bacterial community structure in 17 sputum samples from five CF patients. *Staphylococcus* was detected in fewer samples when DNA was extracted using a standard bacterial lysis method compared to when DNA was extracted using a lysis buffer amended with lysostaphin and lysozyme. The standard lysis method resulted in significantly lower measures of *Staphylococcus* relative abundance and higher levels of community diversity, richness, and evenness compared to the lysostaphin-lysozyme modified method. Measures of community dynamics in serial sputum samples from the same individual were nevertheless highly concordant between the two DNA extraction methods. These results illustrate the impact of DNA preparation method on measures of *Staphylococcus* abundance and bacterial community structures in studies of the airways microbiota in CF.

## Introduction

Cystic fibrosis (CF), an autosomal recessive genetic disease affecting more than 70,000 people worldwide, is caused by mutations in the CF transmembrane conductance regulator gene. The associated alterations in respiratory tract mucociliary clearance and airway surface fluid composition result in chronic infection and inflammation of the airways, leading to progressive lung disease and respiratory failure [1]. Conventional culture-based microbiology has identified a number of bacterial species that commonly infect the airways of persons with CF, including *Pseudomonas aeruginosa*, *Staphylococcus aureus*, *Burkholderia cepacia* complex, *Stenotrophomonas maltophilia*, and *Achromobacter xylosoxidans* [2].

Among these species, *S. aureus* typically infects patients at an early age, being most frequently isolated from infants and children with CF [2], [3]. Recent studies using culture-independent approaches to assess the microbiota of CF airways reiterate that this species is a common constituent of the bacterial communities typically found in CF [4], [5], [6], [7]. As such, it is reasonable to expect that the abundance of *Staphylococcus* in airway communities would have significant bearing on measures of bacterial community structure and dynamics. However, the isolation of *Staphylococcus* DNA is hampered by the rigid cell wall that is characteristic of Gram positive bacteria. In preliminary experiments assessing the airways microbiome in CF, we found that the combination of lysostaphin and lysozyme increased the yield of *Staphylococcus* DNA from sputum samples compared with a standard bacterial cell lysis buffer. We therefore wished to further examine the impact of DNA preparation method on pyrosequencing-based measures of *Staphylococcus* abundance and overall bacterial community structure in sputum specimens from patients culture-positive for *S. aureus*.

## Results

### Impact on *Staphylococcus* prevalence, relative abundance and community diversity, richness and evenness

Seventeen sputum samples from five CF patients were selected for study. *S. aureus* had been cultured from each sample, with recovery graded as rare, few, moderate or numerous by the University of Michigan Health System clinical microbiology laboratory (Table 1). DNA was purified in duplicate from each sputum sample: one preparation used a standard lysis buffer while the other used the same buffer with the addition of lysostaphin-lysozyme. All 34 DNA preparations were subjected to bar-coded pyrosequencing of a portion of the 16S rRNA V3–V5 region using protocols developed for the Human Microbiome Project; sequences were analyzed using the software package mothur v1.21 [8] (see Materials and Methods for details).

**Table 1:** Culture and pyrosequencing based measures of *Staphylococcus* abundance.

Patient	Sputum	Culture^1^	Relative Abundance^2^		Total Taxa^3^		*Staph* Rank^4^	
			−LY	+LY	−LY	+LY	−LY	+LY
1	1	Numerous	0.75	0.98	14	4	1	1
	2	Numerous	0.43	0.9	15	10	1	1
	3	Numerous	0.5	0.86	11	6	1	1
	4	Few	<0.01	<0.01	20	11	11	11
	5	Numerous	0.13	0.73	20	16	2	1
2	6	Numerous	0.04	0.48	32	29	6	1
	7	Numerous	0.08	0.58	25	20	6	1
	8	Numerous	0.2	0.68	17	17	3	1
	9	Numerous	0.3	0.76	18	12	2	1
	10	Numerous	0.21	0.75	19	11	2	1
	11	Numerous	0.04	0.37	18	17	5	1
3	12	Moderate	0	<0.01	4	10	ND	12
	13	Rare	0	<0.01	3	7	ND	6
	14	Rare	<0.01	<0.01	23	20	28	28
	15	Rare	0	0	7	4	ND	ND
4	16^5^	Numerous	<0.01	0.02	34	31	35	6
5	17	Rare	0	<0.01	22	21	ND	17

1 Abundance of *S. aureus* in culture (reported by clinical microbiology laboratory).

2 Relative abundance by pyrosequencing without (−LY) and with (+LY) lysostaphin-lysozyme; no. *Staphylococcus* sequence reads/no. total sequence reads.

3 Total number of OTUs observed after normalization of sequence reads to 498, the smallest number of sequences obtained among the 34 samples.

4 The rank order of the relative abundance of *Staphylococcus*.

5 With the exception of sample 16, samples with “numerous *S. aureus*” detected in culture are referred to as *Staphylococcus*-rich samples. Sample 16 and all other samples are referred to as S*taphylococcus*-poor samples.

ND: not detected.

16S rRNA gene sequences corresponding to *Staphylococcus* were retrieved from all sputum samples that had been graded as having numerous, moderate or few *S. aureus* in culture, irrespective of DNA preparation method (Table 1). However, *Staphylococcus* was detected in none of the four sputum samples graded as having rare growth of *S. aureus* in culture when DNA was prepared using the standard lysis buffer. In contrast, *Staphylococcus* was detected in three of these four sputum samples when DNA was prepared using the lysostaphin-lysozyme amended buffer.

In the six sputum samples that were graded as having moderate, few or rare growth of *S. aureus* in culture, the relative abundance of *Staphylococcus* was <1%, regardless of DNA preparation method (Table 1). In one of the 11 sputum samples graded as having numerous *S. aureus* in culture (sample 16), the relative abundance of *Staphylococcus* was <1% in the sample prepared with standard lysis buffer and 2% in the respective sample prepared with the lysostaphin-lysozyme amended buffer. Together, these seven sputum samples are hereafter referred to as “*Staphylococcus* -poor” samples.

**Figure 1:**
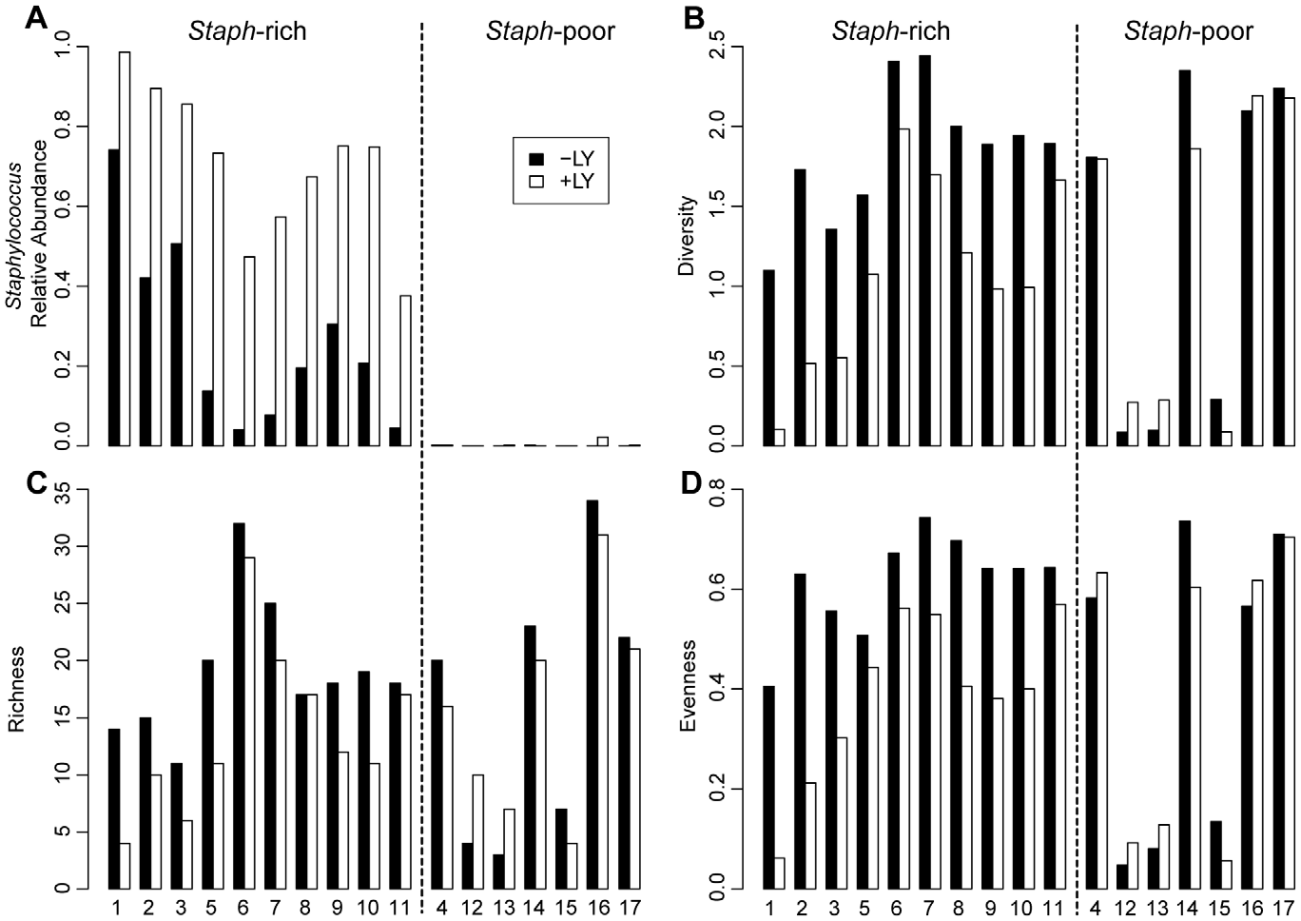
Impact of DNA extraction method on measures of *Staphylococcus* relative abundance alpha diversities. (**A**) Relative abundance of *Staphylococcus* and (**B**) community diversity (Shannon index), (**C**) richness (number of observed OTUs) and (**D**) evenness (Shannon evenness) in *Staphylococcus*-rich and *Staphylococcus*-poor samples revealed by the standard lysis method (−LY, black bars) and the lysostaphin-lysozyme (+LY, white bars) method.

In each of the remaining 10 “numerous *S. aureus* in culture” sputum samples (hereafter referred to as “*Staphylococcus* -rich” samples), the relative abundance of *Staphylococcus* in the samples prepared with the lysostaphin-lysozyme amended lysis buffer (71%; range 37%–98%) was *s*ignificantly higher than that in the respective sample prepared with standard lysis buffer (27%; range 4%–75%; p<0.01, paired t-test) (Table 1 and Figure 1A). For these 10 sputum samples, measures of bacterial community diversity, richness (total number of taxa), and evenness (relative abundance of taxa) were significantly higher in DNA samples prepared with standard lysis buffer compared with DNA samples prepared with lysostaphin-lysozyme amended buffer (Table 1, Figure 1B, 1C and 1D; p<0.05, paired t-test). In contrast, these measures showed little difference between DNA preparation methods in the seven pairs of *Staphylococcus* -poor samples (p>0.05, paired t-test).

Predictably, the increased relative abundance of *Staphylococcus* in the samples prepared with lysostaphin-lysozyme resulted in the decreased relative abundance of other taxa in these samples (ie, compared to the same sample prepared with standard lysis buffer). Consequently, some taxa that were found in low abundance (mean relative abundance 0.4%; range 0.2%–2.4%) in samples prepared with standard lysis buffer were undetectable in samples prepared with lysostaphin-lysozyme. For example, an OTU representing *Prevotella* was detected in all 5 samples from Patient 1 when prepared with the standard lysis buffer, but in only 3 of these 5 samples when prepared with the lysostaphin-lysozyme amended lysis buffer. Similarly, an OTU representing *Fusobacterium* was detected in all 6 samples from Patient 2 when prepared with standard lysis buffer, but in only 4 samples prepared with the amended buffer. In no cases did the increase in relative abundance of *Staphylococcus* result in failure to detect *Pseudomonas* in samples prepared with the amended lysis buffer.

### Impact on community structures

**Figure 2:**
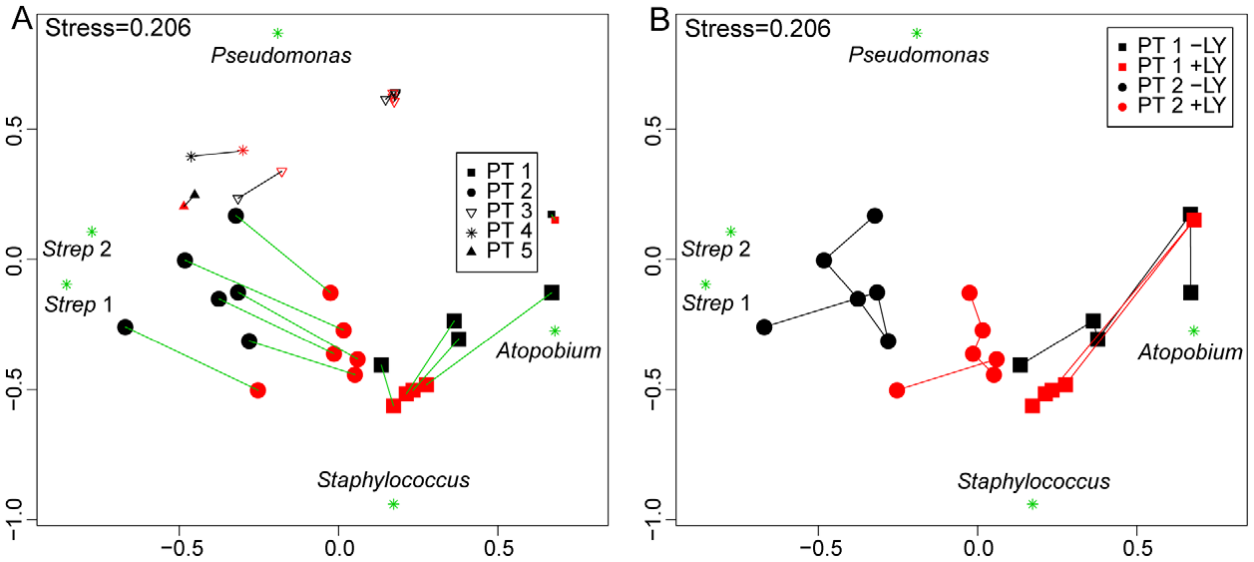
Impact of DNA extraction method on community structure and dynamics. (**A**) Biplot showing the pairwise comparison of community structures for the 10 *Staphylococcus*-rich (large symbols) and the 7 *Staphylococcus*-poor (small symbols) sputum samples using nonmetric multi-dimensional scaling (nMDS). Pairs from the same sample processed with standard lysis method (black symbols) or lysostaphin-lysozyme method (red symbols) are connected by a line and are more similar to each other the closer they are on the ordination plot. The top 5 OTUs (labeled as green stars) most responsible for shifting samples on the ordination plot were: *Staphylococcus*, *Pseudomonas*, *Strep 1* (*Streptococcus pneumoniae*), *Strep 2* (*Streptococcus milleri* group) and *Atopobium*. (**B**) Comparison of community structure dynamics in serial sputum samples collected from Patient 1 (square, n = 5) and Patient 2 (circles, n = 6). The ordination plot is the same as that in (A) except that samples prepared by the standard lysis method (black) or lysostaphin-lysozyme method (red) are connected by lines chronologically.

In addition to the effects on measures of alpha diversity, DNA extraction method also had a marked effect on measures of beta diversity among the *Staphylococcus*-rich samples. This was evident in the Bray-Curtis distances between communities observed in each member of the 17 pairs of DNA samples. The mean Bray-Curtis distance within pairs of DNA samples from the 10 *Staphylococcus* -rich sputum samples (0.46) was significantly higher than the mean distance within pairs of DNA samples from the seven *Staphylococcus* -poor sputum samples (0.13) (p<0.01, two-tailed t-test). Bray-Curtis distance-based nonmetric multi-dimensional scaling (NMDS) highlighted these differences (Figure 2A). DNA extraction method had little effect on the *Staphylococcus*-poor samples, with paired communities residing close to each other on the ordination plot. In contrast, among the *Staphylococcus*-rich samples, communities in samples extracted with lysostaphin-lysozyme were more separated from their respective communities in samples prepared with the standard DNA extraction method. NMDS indicated that these shifts were driven by *Staphylococcus* (Figure 2A).

### Impact on community dynamics

To investigate the impact of DNA extraction method on bacterial community dynamics, Bray-Curtis distances between five sputum samples obtained from Patient 1 during a 15 month period and six sputum samples obtained from Patient 2 during a 50 month period were assessed using NMDS (Figure 2B). The two DNA extraction methods resulted in similar community structure dynamics in both patients, as reflected by similar movements on the ordination plot. Procrustes analysis, a statistical shape analysis approach, confirmed that the movement of samples on the ordination plot revealed by the two methods were highly correlated (r = 0.83, p = 0.001). These correlations were also shown by the Mantel test comparing Bray-Curtis distance matrices (Patient 1: Mantel r = 0.77, p = 0.04; Patient 2: Mantel r = 0.91, p<0.01).

## Discussion

Previous studies of *Staphylococcus* have enhanced bacterial cell lysis by the addition of various reagents to extraction buffers, including lysostaphin, proteinase K, and/or detergents such as sodium dodecyl sulfate (SDS) [9]. In this study we chose the combination of lysostaphin and lysozyme, which provided considerably greater yield of *Staphylococcus* DNA than did the use of a standard lysis buffer in preliminary experiments. Although the impacts of this modification on the detection of *Staphylococcus* and measures of the relative abundance of this and other taxa in CF sputum samples are apparent and predictable, these findings have important implications for studies of the CF airway microbiota.

Zemanick and colleagues [10] recently found that culture was more sensitive in detecting *S. aureus* in CF respiratory specimens than was a species-specific quantitative PCR (qPCR) assay. DNA was extracted from specimens with a buffer containing 5.7% SDS, but no lysostaphin or lysozyme. For specimens with relatively low quantities of *S. aureus* (10^3^–10^5^ cfu/ml) in culture, the sensitivity of qPCR for detecting this species was only 22%; in specimens with greater quantities of *S. aureus* (>10^5^ cfu/ml) in culture, the sensitivity of qPCR was 87%. We similarly found a lower rate of detection of *Staphylococcus* using a culture-independent approach compared to culture. With a standard DNA extraction protocol, 13 (76%) of 17 *S. aureus* culture-positive specimens yielded pyrosequencing reads classified as *Staphylococcus*. However, with the addition of lysostaphin and lysozyme to the DNA extraction buffer, the sensitivity of pyrosequencing in detecting *Staphylococcus* increased to 94%.

We further investigated the impact of DNA extraction method on overall measures of bacterial community structure in CF sputum. The addition of lysostaphin-lysozyme resulted in measures of greater *Staphylococcus* relative abundance and lowered measures of the relative abundance of other taxa, as well as estimates of overall species richness. Considering the prominent role that *S. aureus*, including methicillin-resistant *S. aureus*, is believed to play in CF lung disease [2], [11] these findings deserve careful consideration when designing studies using culture-independent approaches to investigate the microbiome of the CF airways. This is especially relevant in studies involving children insofar as the prevalence of *S. aureus* infection in CF is highest among patients aged 6 to 10 years [3]. Mild pulmonary disease and lack of sputum production are common in this age group, necessitating the use of throat swab cultures to assess airway bacterial communities. Relative to culture, the sensitivity of culture-independent methods to detect *S. aureus* is considerably lower with oropharyngeal swab specimens than with expectorated sputum samples [10].

In summary, DNA preparation method must be taken into account when considering studies describing rates of *Staphylococcus* detection based on culture-independent methods. Enhancing *Staphylococcus* cell lysis and DNA extraction would be desirable in studies in which determining the prevalence and/or relative abundance of *Staphylococcus* is important. In contrast, in studies focusing on the correlation between specific taxa, particularly low abundance species, and disease or when more accurate estimates of total microbial community richness are sought, decreased estimates of the relative abundance of *Staphylococcus* may be warranted. Importantly, despite the marked effect of DNA extraction method on alpha and beta diversities, especially in *Staphylococcus*-rich samples, the community dynamics revealed by the two methods are highly correlated.

## Materials and Methods

### Ethics Statement

This study was approved by the University of Michigan Institutional Review Board (HUM00003517). As this study was limited to a retrospective analysis of existing unique-identifier-encoded (to protect patient anonymity) biologic specimens and clinical data, waiver of subject informed consent was granted.

### Sputum samples

Sputum specimens, collected from CF patients during the course of routine care, were obtained from the University of Michigan Health System clinical microbiology laboratory. After removing an aliquot of the sample for bacterial culture, the remainder of the unadulterated sample was recovered, divided into 0.5 ml aliquots, and stored at −80°C until processing for DNA extraction.

### DNA extraction

Samples from frozen stock were thawed on ice and incubated with an equal volume of 10% Sputolysin (EMD Chemicals, Gibbstown, NJ) at 37°C for 30 min with pulse vortexing at 5 min intervals. Samples that were not of uniform consistency after 30 min were mechanically homogenized for 10 sec with a tissue homogenizer (Omni International, Kenneshaw, GA). Samples were then mixed with 0.9 volume of MagNA Pure Bacterial Lysis Buffer (Roche Applied Science, Indianapolis, IN) either with or without the addition of lysostaphin (final concentration 0.14 mg/ml, Sigma-Aldrich Corp., St. Louis, MO) and lysozyme (final concentration 2.9 mg/ml, Sigma-Aldrich Corp.). Lysostaphin-lysozyme containing preparations were incubated another 30 min at 37°C. All samples were then transferred to microfuge tubes containing garnet beads (MO BIO Laboratories, Inc., Carlsbad, CA) and agitated in a Mini-Beadbeater-8 (Biospec Products Inc., Bartlesville, OK) for 1 min on maximum setting. After digesting with Proteinase K (final concentration 1.4 mg/ml) for 10 min at 65°C, samples were again agitated in the Beadbeater and incubated at 95°C for an additional 10 min. DNA was purified using a MagNA Pure Compact System (Roche) according to the manufacturer's DNA Bacteria v3.1 protocol. As a negative control, the same procedure was used with sterile water replacing sputum in each extraction; no PCR products were detected in any experiment, indicating lack of contamination of any of the reagents used.

### Bar-coded pyrosequencing

DNA pyrosequencing was performed by the Human Genome Sequencing Center at Baylor College of Medicine. The V3, V4 and V5 hypervariable regions of the 16S rRNA gene were amplified using primer 357F (5′-CCTACGGGAGGCAGCAG-3′) modified with the addition of the 454 FLX-titanium adaptor “B” sequence (5′-CCTATCCCCTGTGTGCCTTGGCAGTCTCAG-3′) and primer 926R (5′-CCGTCAATTCMTTTRAGT-3′) modified with the addition of unique 6–8 nucleotide barcode sequences and the 454 FLX-titanium adaptor “A” sequence (5′-CCATCTCATCCCTGCGTGTCTCCGACTCAG-3′). Barcode and adaptor sequences are found at http://www.hmpdacc.org/doc/HMP_MDG_454_16S_Protocol_V4_2_102109.pdf. PCR amplification was performed on 2 µl of DNA template in a total volume of 25 µl containing 1× AccuPrime Buffer II (Invitrogen Corp., Carlsbad, CA), 320 µM of each primer, and 0.03 U/µl AccuPrime High Fidelity *Taq* polymerase. Reactions were heated at 95°C for 2 min followed by 30 cycles of 95°C for 20 sec, 50°C for 30 sec, and 72°C for 5 min. The concentration of amplicons in each reaction was determined in triplicate using the PicoGreen fluorescent assay (Invitrogen Corp.), and amplicons were pooled in groups of 96 in equal proportion before being sequenced via 454 FLX-titanium according to manufacturer's specifications. A water sample was included in each pyrosequencing run as a negative control.

### Sequence processing and statistical analysis

Raw sequences were analyzed using mothur v1.21 [8] to remove sequences containing homopolymers greater than 8 bp, mismatches in the barcode or primer, one or more ambiguous bases, or an average quality score below 35 over a moving window of 50 bp. Remaining sequences that were at least 200 bp but less than 590 bp in length were further curated to remove chimeric sequences using UCHIME [12] and to reduce sequencing noise by a preclustering methodology [13] before being assigned to operational taxonomic units (OTUs) using an average neighbor algorithm with a 0.03 dissimilarity cutoff. The consensus taxonomy of each OTU was identified at the genus level using the Bayesian method [14]. The total number of reads for each community was normalized to 498, the smallest number of reads among the samples included in the study, to control for differences in sequencing depth before alpha and beta diversity measures were calculated. Community diversity was measured using non-parametric Shannon indices [15]. The number of observed OTUs was used as a measure of community richness. Community evenness was measured with Shannon indices-based measure of evenness. Beta diversity was measured using Bray-Curtis dissimilarity coefficients.

Procrustes analysis was used to compare the correlation of NMDS configurations by scaling and rotating one to the other. The statistical significance of the correlation between the two configurations was calculated by a permutation procedure (protest function) [16]. The Mantel test, used to test the correlation between Bray-Curtis based community measures, was performed with 10000 permutations measuring the Spearman correlation. Both the Procrustes analysis and the Mantel test were performed using the R package vegan (http://CRAN.R-project.org/package=vegan). Paired t-test was used to compare the relative abundance of *Staphylococcus*, and community diversity, richness, and evenness between sets of samples. Two-tailed t-test was performed to compare the average Bray-Curtis distances between the *Staphylococcus*-rich and *Staphylococcus*-poor samples. A p<0.05 was considered statistically significant.
